# Lymph node metastases >5 and metastatic lymph node ratio >0.30 of differentiated thyroid cancer predict response to radioactive iodine

**DOI:** 10.1002/cam4.4288

**Published:** 2021-10-08

**Authors:** Canhua Yun, Juan Xiao, Jingjia Cao, Chunchun Shao, Lihua Wang, Wei Zhang, Hongying Jia

**Affiliations:** ^1^ Department of Nuclear Medicine The Second Hospital Cheeloo College of Medicine Shandong University Jinan China; ^2^ Center of Evidence‐Based Medicine The Second Hospital Cheeloo College of Medicine Institute of Medical Sciences Shandong University Jinan China; ^3^ School of Public Health Cheeloo College of Medicine Shandong University Jinan China

**Keywords:** classification tree, differentiated thyroid cancer, LNMs, LNR, radioactive iodine

## Abstract

**Purpose:**

The study was designed to elucidate the predictive value of the number of lymph node metastases (LNMs) and lymph node ratio (LNR) for response to therapy restratification system (RTRS).

**Methods:**

From December 2015 to December 2019, 1228 patients who accepted radioactive iodine (RAI) were collected in the study. After 6–8 months, response to RAI was evaluated as complete response (excellent response) and incomplete response (indeterminate, biochemical, and structural incomplete response). The study developed classification tree to determine the optimum LNMs and LNR that predicted response to RAI. Multivariate logistic regression analyses were further analyzed to find independent factors of response to RAI.

**Result:**

The mean age of patients was 44 ± 12 and 71.09% (873/1228) were females. The best cutoff value of LNMs to affect RAI treatment response determined by classification tree was 5. Further in 388 patients with LNMs >5, the best cutoff value of LNR to affect RAI treatment response determined by classification tree was 0.30. With multivariate analysis, the study found that LNMs (>5), gender, lymph node dissection, and American Thyroid Association (ATA) risk classification were independent predictors of response to RAI for all 1228 patients; and LNR (>0.30), gender, and ATA risk classification for 388 patients with LNMs >5. The sensitivity analysis indicated that whether patients with LNM or not were included, the multivariate logistic regression model was kept stable. On subgroup analysis, no significant interactions were observed between the effect of LNMs/LNR and gender, N stage, ATA risk classification, lymph node dissection, or T stage.

**Conclusions:**

With classification tree, the study found that LNMs and LNR could predict initial response to RAI, and their optimal cutoff values were 5 and 0.30, separately.

## Graphical abstract

The prognostic significance of lymph node metastasis for DTC patients remains controversial. With classification tree, the study found increasing LNMs and LNR may be risk factors for initial RAI therapy response, and optimal cutoff values were 5 and 0.30, separately.

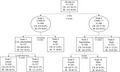

## 1 INTRODUCTION

In recent years, the incidence rate of differentiated thyroid cancer (DTC) has increased rapidly.^1^ Lymph node metastases (LNMs) were found in 30%–80% of DTC patients.^2^ For patients with LNM, postoperative radioactive iodine (RAI) is an effective treatment. Since DTC can active uptake iodine as thyrocyte and a key target of RAI is a functional sodium iodide symporter that captures iodine and its intracellular transport. So, RAI can effectively eliminate remnant cancer cells. Today, postoperative RAI with ^131^I for DTC patients is widely used worldwide.^3^

The extent of lymph node involvement is an essential part to evaluate patients’ condition and formulate treatment plan of RAI, which is a key part of 2015 American Thyroid Association (ATA) initial risk stratification system.^4^ The number of LNM (LNMs) is a common indicator for evaluating lymph node involvement. However, the prognostic significance of it for DTC patients remains controversial. Another indicator for LNM, lymph node ratio (LNR), which indicate the involved ratio of metastatic lymph nodes, can represent degree of surgical removal of the involved LNM. It remains uncertain whether LNR holds values for outcomes in DTC patients.^5^, ^6^ Moreover, most previous studies regarded the recurrence or mortality of patients as outcomes,^7^, ^8^ but lacked evaluation of response to initial RAI therapy.

In this study, we applied the response to therapy restratification system (RTRS)^9^ to evaluate DTC patients’ response to initial RAI therapy. With the method of classification tree, the study was designed to elucidate optimal cutoff values of LNM/LNR to predict for RTRS.

## 2 MATERIALS AND METHODS

The patient data extracted from clinical electronic medical record (EMR) were confirmed not to include all personal information. The Institutional Review Board (IRB) of the “Blinded to peer review” approved this study. All patients signed informed consent before initial RAI. All procedures complied with the Declaration of Helsinki for research involving human subjects.

### 2.1 Study population

From December 2015 to December 2019, 1771 consecutive patients who accepted RAI were initially enrolled. All patients underwent (1) evaluation of preoperative cervical physical examination and ultrasonography for primary tumors and loco‐regional lymph nodes and (2) total thyroidectomy and prophylactic bilateral central (compartment VI) node dissection before presentation for RAI. Whether to dissect lateral cervical lymph nodes was decided by surgeons according to the preoperative ultrasound and fine‐needle aspiration (FNA). The inclusion criteria were as follows: (1) pathologic type was papillary/follicular thyroid carcinoma; (2) initial RAI without a history of RAI therapy; and (3) information available of pathological information including primary tumors and LNM, details of surgery, and medical examination just before RAI therapy. Among them, 543 patients were excluded for the following reasons: (1) initial distant metastases found before initial RAI; (2) follow‐up information was insufficient after initial RAI therapy; and (3) patients had recent neck irradiation within 3 months before RAI. Finally, a total of 1228 patients were included in the study.

### 2.2 Initial RAI Therapy

RAI therapy started at least 4 weeks after surgery. Patients were asked to take a low‐iodine diet with thyroid hormone withdrawal (THW) for a month prior to RAI. Routine medical examinations included thyroid‐stimulating hormone (TSH), pre‐ablation stimulated thyroglobulin (ps‐Tg), thyroglobulin antibody (TgAb), and neck ultrasound (US) were performed just before initial RAI. TSH >30 μIU/ml was required after THW. Doses of RAI ranged from 3.70 GBq (100 mCi) to 5.55 GBq (150 mCi) were delivered according to 2015 ATA risk stratification. A whole‐body post‐treatment scan was performed 72h after RAI. Patients were then discharged with TSH‐suppressive dose of L‐Thyroxine 4, 125–175 μg orally per day according to ATA risk classification for recurrence.

### 2.3 Follow‐up protocol

After initial RAI therapy, patients received regular follow‐up with serum biochemical tests including TSH, TSH‐stimulated/TSH‐suppressed Tg, free triiodothyronine, free thyroxine, TgAb, and neck ultrasound per 1 month for first 3 months and then every 6 months. Diagnostic whole‐body scan (DxWBS) was performed 6–8 months after initial RAI.

### 2.4 Tumor–node–metastasis (TNM) staging

Based on pathology reports, patients were evaluated to match the eighth edition of the AJCC/TNM staging system of thyroid cancer^25^.

### 2.5 ATA risk classification

ATA risk classification of patients was according to the ATA guideline (2015).^4^

### 2.6 Definitions of Response to RAI therapy

Combining the serological results and image findings 6–8 months after initial RAI therapy, the study used RTRS recommended by the 2015 ATA guideline^4^ to assess the patients’ condition. Response to RAI therapy was classified into excellent response (ER), biochemical incomplete response (BIR), structural incomplete response (SIR), and indeterminate response (IDR). In the study, the four responses to RAI were divided into two groups, complete response (CR) (included ER) and incomplete response (IR) (included IDR, BIR, and SIR).

### 2.7 Statistical analysis

Numerical variables were expressed as means±standard deviations (*SD*), or medians with interquartile ranges (*P_25_*, *P_75_*). Independent *t*‐test was used to compare the difference in numerical variables with normal distribution and Wilcoxon rank‐sum test was used for numerical variables that did not obey normal distribution. Categorical variables were expressed as numbers (percentages). Pearson's Chi‐squared test or Fisher's exact test (if applicable) was used to compare differences in categorical variables. The study developed classification tree to determine the optimum LNMs (≤5 and >5) that predicted response to RAI, so patients were classified into low LNMs as ≤5 and high LNMs as >5. The exhaustive CHAID method was used to build the classification tree model. The depth of the model was automatically selected. The minimum number of parent node was 40 and the minimum number of child node was 20. The box represented the terminal node and the ellipse represented the non‐terminal node. LNR was defined as LNMs divided by the number of removed lymph nodes. Considering the control for low lymph node yields which tended the LNR to extremum, we only calculated LNR in high LNMs group (>5 LNMs). Classification tree showed that 0.30 is the optimal cutoff value of LNR. So, we defined low LNR group as ≤0.30 and high LNR group as >0.30. Multivariate logistic regression analyses were further analyzed to find LNMs, LNR, and other independent factors associated with response to RAI. First, univariate analysis was used, and then the significant variables at 0.10 (*α*) level were included in the multivariate logistic model. Based on the Akaike Information Criterion (AIC), the model is simplified by bidirectional stepwise elimination approach. The variance inflation factor (VIF) was used to measure multicollinearity. VIF >10 was the standard to judge the multicollinearity of factors. To investigate the relationship between LNMs and response to RAI more thoroughly and comprehensively, a sensitivity analysis was performed only in patients with metastatic lymph nodes, to repeatedly explore the association between LNMs and response to RAI.

All tests were bilateral, and *p* value <0.05 was considered statistically significant. The software package R (version 3.5.3) and SPSS version 22.0 were used for statistical analysis.

## 3 RESULTS

### 3.1 Clinicopathological features of patients

Among 1228 DTC patients, the mean age was 44 ± 12 years old and 71.09% (873/1228) were females. Among all the patients, 615 (50.08%) were classified into the CR group (ER, *n* = 615) and 613 (49.92%) into the IR group (IDR, *n* = 194; BIR, *n* = 105; SIR, *n* = 314). Of these patients, 12.38% (152/1228) had no LNM, 48.94% (601/1228) was in the stage of N1a, and 38.68% (475/1228) was in the stage of N1b.

Table 1 shows the univariate analysis of clinicopathological characteristics and response to therapy groups for the initial RAI therapy. The levels of LNMs in IR group were higher than that in CR group (*p* < 0.001). The proportion of IR was also higher in male group (*p* < 0.001), lateral and central node dissection group (*p *< 0.001), high level group of Tumor Stage (*p *= 0.003), N1b group (*p *< 0.001), and high level of ATA risk classification (*p *< 0.001).

**TABLE 1:** Clinicopathological characteristics stratified by response to therapy.

Variables	Total (*n* = 1228)	Complete response (*n* = 615)	Incomplete response (*n* = 613)	*p* value
Age at diagnosis^a^	44 ± 12	42 + 11	43 + 12	0.140
LNMs^b^	3.00 (1.00, 7.00)	3.00 (1.00, 5.00)	4.00 (2.00, 8.00)	**<0.001**
Gender^c^				**<0.001**
Female	873(71.09)	478 (77.72)	395 (64.44)	
Male	355 (28.91)	137 (22.28)	218 (35.56)	
Lymph node dissection^c^				**<0.001**
Lateral and central node dissection	768 (62.54)	345 (56.10)	423 (69.01)	
Central node dissection	460 (37.46)	270 (43.90)	190 (31.00)	
Tumor stage^c^				**0.003**
T0	3 (0.24)	3 (0.49)	0 (0.00)	
T1a	526 (42.83)	285 (46.34)	241 (39.32)	
T1b	383 (31.19)	201 (32.68)	182 (29.69)	
T2	158 (12.87)	66 (10.73)	92 (15.01)	
T3a	17 (1.38)	6 (0.98)	11 (1.79)	
T3b	23 (1.87)	9( 1.46)	14(2.28)	
T4a	4 (0.33)	2 (0.33)	2(0.33)	
Tx	114 (9.28)	43 (6.99)	71 (11.58)	
Node stage^c^				**<0.001**
N0	152 (12.38)	80 (13.00)	72 (11.75)	
N1a	601(48.94)	338 (54.96)	263 (42.90)	
N1b	475(38.68)	197 (32.03)	278 (45.35)	
TNM stage^c^				0.181
I	1035 (84.28)	529 (86.02)	506 (82.55)	
II	189 (15.39)	85 (13.82)	104 (16.97)	
III	4 (0.33)	1 (0.16)	3 (0.48)	
ATA risk classification^c^				**<0.001**
Low risk	129 (10.50)	76 (12.36)	53 (8.65)	
Intermediate risk	927 (75.49)	518 (84.23)	409 (66.72)	
High risk	172 (14.01)	21 (3.41)	151 (24.63)	

Abbreviations: LNMs, number of lymph node metastases; ATA, American Thyroid Association.

a means±standard deviations

b medians with interquartile ranges (*P_25_*, *P_75_*)

c numbers (percentages).

The bold values meaned *P* < 0.05, which showed statistically significance.

The clinicopathological characteristics of the whole cohort stratified by response to therapy are shown in Table 1.

### 3.2 The best cutoff value of LNMs for initial RAI response determined by classification tree

**FIGURE 1:**
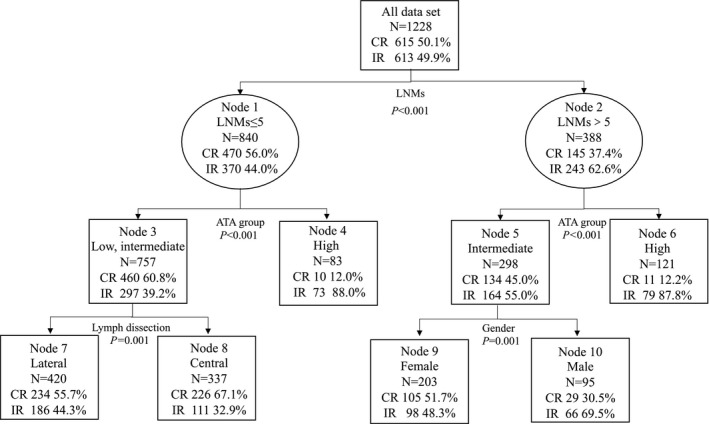
The classification tree for determining the best cutoff value of LNMs to RAI treatment response

In the univariate analysis, the significant variables at 0.10 (*α*) level were gender, LNMs, tumor stage, node stage, ATA risk classification, and lymph node dissection. Since it was found multicollinearity using VIF between lymph node dissection and node stage, so the study selected “lymph node dissection” according to the index of AIC into the classification tree. So, finally the classification tree included LNMs, gender, lymph node dissection, and ATA risk classification. The tree model consisted of three layers: the first layer was LNMs (cutoff value = 5, *p *< 0.001), the second layer was ATA group, and the third layer was lymph dissection and gender. The predictive accuracy of the model was 74.9% (standard error was 0.025). The best cutoff value of LNMs to affect RAI treatment response determined by classification tree was 5. In the study, 30.12% (388/1288) of patients were detected with more than five LNMs, as high LNMs group, whereas the remaining 69.88% (840/1288) of patients had ≤5 LNMs, as low LNMs group. Details are shown in Figure 1.

### 3.3 Multivariate logistic regression analysis in all 1228 patients

In multivariate analysis, we found that LNMs, gender, lymph node dissection, and ATA risk classification were independent factors of response to RAI. Compared to LNMs ≤5, LNMs >5 was a risk factor for IR, *OR* = 1.627 (95%*CI*: 1.236–0.144, *p *< 0.001). Moreover, compared with men, women's ablation was more effective, *OR* = 0.543 (95%*CI*: 0.416–0.709, *p *< 0.001). Lymph node dissection and ATA risk classification were also independent risk factors for IR. Details are shown in Table 2.

**TABLE 2:** Univariate and multivariate binary logistic regression of associated factors for incomplete response (IR) after RAI in 1228 patients

Variables	Univariate analysis			Multivariate analysis		
	*Β*	*OR* (95%*CI*)	*p* value	*β*	*OR* (95%*CI*)	*p* value
LNMs						
≤5	Ref	Ref	.	Ref	Ref	.
> 6	0.756	2.129 (1.665, 2.728)	**<0.001**	0.487	1.627 (1.236, 2.144)	**<0.001**
Gender						
Male	Ref	Ref	.	Ref	Ref	.
Female	−0.655	0.519 (0.403, 0.667)	**<0.001**	−0.610	0.543 (0.416, 0.709)	**<0.001**
Lymph node dissection						
Central node dissection	Ref	Ref	.	Ref	Ref	
Lateral and central node dissection	0.555	1.742 (1.380, 2.203)	**<0.001**	0.445	1.561 (1.210, 2.017)	**0.001**
Node stage						
N0	Ref	Ref	.			
N1a	−0.146	0.865 (0.605, 1.237)	0.424			
N1b	0.450	1.568 (1.087, 2.266)	**0.016**			
Tumor stage	0.207	1.230 (1.070, 1.415)	**0.004**			
ATA risk classification						
Low risk	Ref	Ref		Ref	Ref	
Intermediate risk	0.169	1.184 (0.812, 1.741)	0.384	−0.051	0.950 (0.640, 1.419)	0.800
High risk	2.378	10.786 (6.138, 19.649)	**<0.001**	2.088	8.065 (4.503, 14.945)	**<0.001**

Note

incomplete response (IR) including IDR+BIR+SIR, LNMs: number of lymph node metastases. ATA risk classification was evaluated according to ATA guideline (2015).

The bold values meaned *P* < 0.05, which showed statistically significance.

### 3.4 Sensitivity analysis for multivariate logistic regression model

A sensitivity analysis was performed in 1076 patients only with LNM to repeatedly construct the same multivariate logistic regression model as above. The *OR* of LNMs for IR was 1.620 (1.229, 2.138), which was not different compared with the *OR*, 1.627 (1.236, 2.144) for all 1288 patients (*p *> 0.05). It indicated that whether patients with LNM or not, the multivariate logistic regression model was kept stable.

### 3.5 The best cutoff value of LNR for initial RAI response determined by classification tree in high LNMs group (LNMs >5)

**FIGURE 2:**
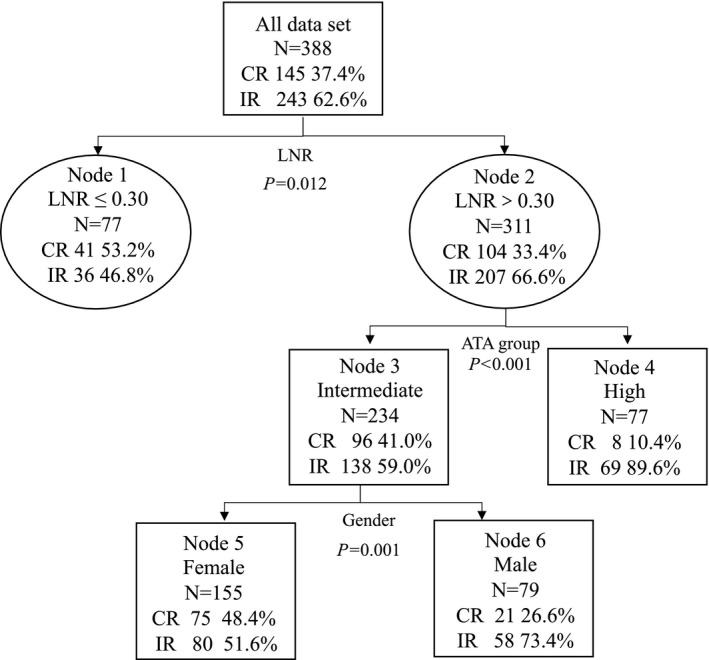
The classification tree for determining the best cutoff value of LNR to RAI treatment response

In 388 patients with more than 5 LNMs, LNR, gender, and ATA risk classification were included into the classification tree. The tree model consisted of three layers: the first layer was LNR (cutoff value = 0.30, *p *= 0.012), the second layer was ATA group (*p *< 0.001), and the third layer was gender (*p* = 0.001). The predictive accuracy of the model was 74.5% (standard error was 0.024). The best cutoff value of LNR to affect RAI treatment response determined by classification tree was 0.30. In the study, 19.85% (77/388) of patients had ≤0.30 LNR, as low LNR group, whereas the remaining 80.15% (311/388) of patients were detected with more than 0.30 of LNR, as high LNR group. Details are shown in Figure 2.

### 3.6 Multivariate logistic regression analysis in 388 patients with LNMs >5

With multivariate logistic regression analysis, LNR, gender, and ATA risk classification were found to be associated with response to RAI. Compared with LNR≤0.30, LNR >0.30 was a risk factor for IR, *OR* = 1.963 (95%*CI*: 1.180–3.280, *p *= 0.010). Moreover, ablation was more effective in females in comparison with males, *OR* = 0.435 (95%*CI*: 0.262–0.710, *p *= 0.001). And ATA risk classification was also a risk factor for IR, *OR* = 6.083 (95%*CI*: 3.191–12.658, *p *< 0.001). Details are shown in Table 3.

**TABLE 3:** Univariate and multivariate binary logistic regression of associated factors for incomplete response (IR) after RAI in 388 patients with >5 LNMs.

Variables	Univariate analysis			Multivariate analysis		
	*β*	*OR* (95%*CI*)	*p* value	*β*	*OR* (95%*CI*)	*p* value
LNR						
≤0.30	Ref	Ref	.	Ref	Ref	.
> 0.30	0.771	2.161 (1.337, 3.503)	**0.002**	0.674	1.963 (1.180, 3.280)	**0.010**
Gender						
Male	Ref	Ref	.	Ref	Ref	.
Female	−0.772	0.462 (0.284, 0.736)	**0.001**	−0.832	0.435 (0.262, 0.710)	**0.001**
ATA risk classification						
Intermediate risk	Ref	Ref		Ref	Ref	.
High risk	1.757	5.794 (3.078, 11.926)	**<0.001**	1.806	6.083 (3.191, 12.658)	**<0.001**

Note

incomplete response (IR) including IDR+BIR+SIR, LNR: lymph node ratio. ATA risk classification was evaluated according to ATA guideline (2015).

The bold values meaned *P* < 0.05, which showed statistically significance.

### 3.7 Subgroup analysis to explore relationships of LNMs/LNR and response to RAI

On subgroup analysis (Appendix Figure 3. and Figure 4), no significant interactions were observed between the effect of LNMs/LNR and gender, N stage, ATA risk classification, lymph node dissection, or T stage.

**FIGURE 3:**
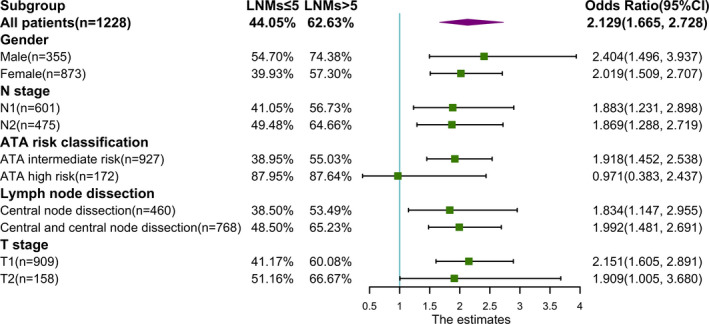
Forest plot of the association between LNMs and RAI treatment response by subgroup. Odds ratio and unadjusted odds ratio are associated with LNMs (LNMs ≤5 is the reference, OR = 1)

**FIGURE 4:**
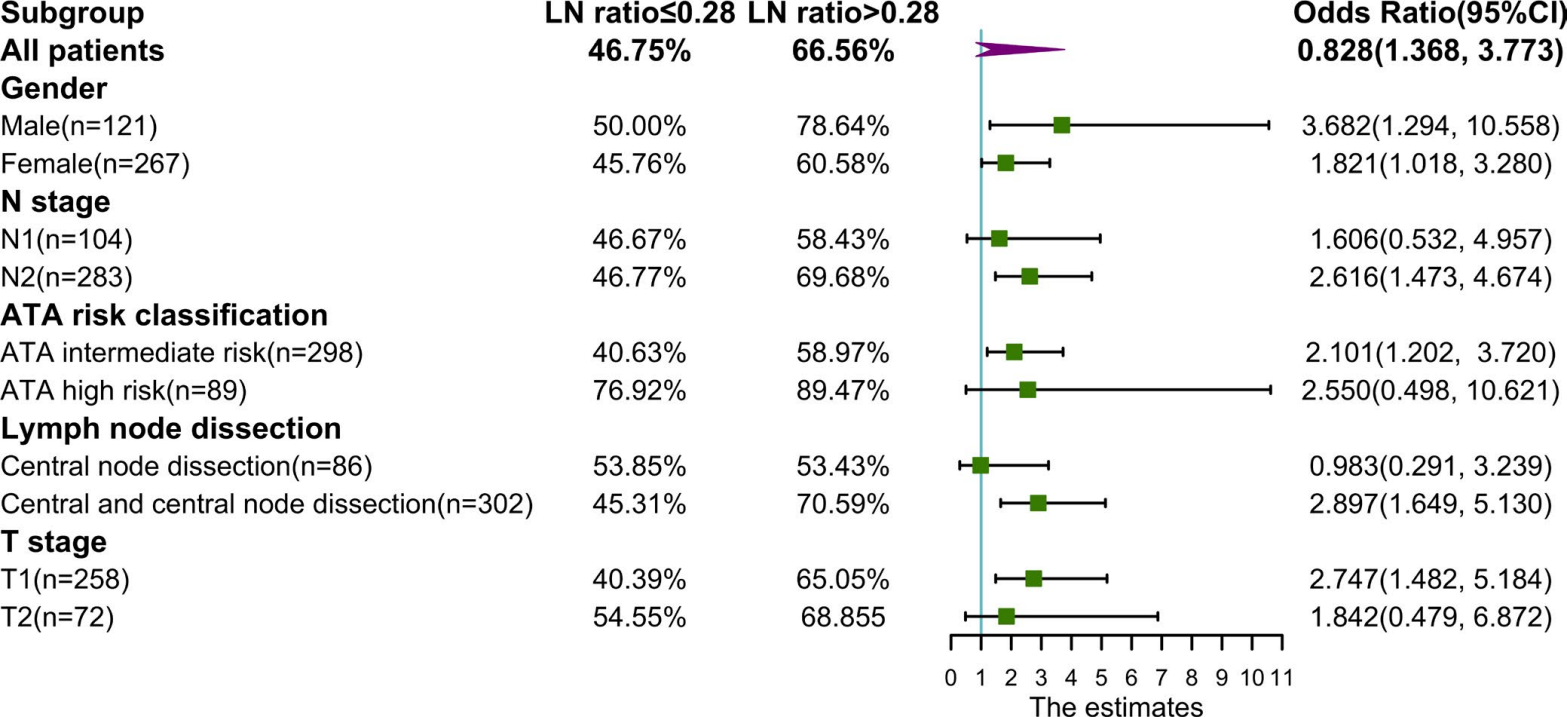
Forest plot of the association between LNR and RAI treatment response by subgroup. Odds ratio and unadjusted odds ratio are associated with LNR (LNR ≤0.30 is the reference, OR = 1)

## 4 DISCUSSION

For DTC patients with intermediate or high risk of recurrence, surgical resection and subsequent RAI therapy are recommended as the first‐line regimen to reduce local recurrence.^10^ It has been reported that LNMs and LNR had statistically significant effects on the prognosis of DTC patients. With the aid of classification tree, the study clarified the best cutoff value of LNMs for RAI response was 5; further, in patients with more than 5 LNMs, the best cutoff value of LNR for RAI response was 0.30. Nowadays, same treatments were used for patients with neck metastases, but the study revealed the response to RAI was extremely different according to LNMs and LNR. We believe that our findings may be of great significance for clinicians to make follow‐up treatment recommendations.

Several studies reported the importance of LNMs in predicting recurrence. Lee et al showed that LNMs were a poor prognostic factor, and recurrence‐free rate was statistically different between patients with 0–1 LNMs and those with 2 or more LNMs.^11^ Study by Ricarte J suggested that the recurrence‐free survival rate of patients with more than three LNMs was significantly lower than that of patients with <3 LNMs.^12^ Sugitani I et al. and Ito et al. reported that the ≥5 LNMs increased the recurrence rate.^13^, ^14^ Other studies have revealed that patients with LNMs >10 were an important risk factor for persistent and recurrent disease.^15^ The 2015 ATA guidelines also specified the LNMs≥5 was an important part to stratify ATA risk classification for recurrence.^4^

Moreover, we showed the prognostic values of LNR on response to RAI. Several studies aimed to explore the relationship between LNR and recurrence or survival in patients with DTC, but the value of LNR showed conflicting evidence. Jeon et al. showed high LNR (defined as higher than 0.4) was a significant risk factor for recurrence of DTC patients.^5^ In Lee et al's study, LNR ≥0.26 was an important risk factor for regional lymph node recurrence (OR = 11.63, *p* = 0.003).^6^ Another study showed LNR >0.3 was an independent factor for recurrence‐free survival.^16^ Multivariable analysis showed that LNR greater than 0.19 was independently associated with poor disease‐specific survival (HR = 4.11; 95%CI, 2.11–8.97) and overall survival (HR, 2.26; 95%CI, 1.12–5.34).^17^ In Schneider's study, according to disease‐specific mortality, LNR ≥0.42 best divided patients with lymph node metastasis.^18^ However, little study explored the relationship between LNMs/LNR and the treatment response to initial RAI.

In former studies, the receiver operating characteristic (ROC) was usually applied to determine the cutoff value of LNMs/LNR.^19^, ^20^ However, when applying ROC, LNMs/LNR is often taken as the only one variable to construct the model, and influences of confounding factors like age, gender, and pathological type are ignored. This would lead to inaccurate estimation of optimal cutoff values. Adam MA et al. used smoothed restricted cubic spline to ascertain the best cutoff value of LNMs for DTC patients’ survival rate.^21^ However, this method requires manual setting the number of spline function nodes that determines the shape and fitting effect of the curve, which may lead to inaccurate model construction. A reliable method is needed to confirm the optimal cutoff value of LNMs/LNR for initial RAI response.

The classification tree is a helpful statistical model, which can deal with multiple independent variables to explore the relationship among them.^22^ The classification tree can provide cutoff values of clinical indicators through Gini index to provide quantitative basis for doctors.^23^, ^24^ With applying classification tree, the study found that the optimal cutoff value of LNMs/LNR for initial RAI therapy response. At the same time, the model could also adjust confound factors like gender, ATA group, and operation mode.

Most former studies only included patients with LNM to make the research proceeding, however, in our study, patients without LNM were also included. For patients without LNM in the study, accepting RAI was due to one of the followings: invasion of tumor into the perithyroidal soft tissues, vascular invasion, incomplete tumor dissection, aggressive histology (e.g., tall cell, columnar cell carcinoma, and hobnail variant), and personal wishes. We performed a sensitivity analysis to examine whether the group of patients without LNM made significant effect on the relationship between LNMs and response to therapy of RAI. It indicated that the multivariate model was kept stable when patients without LNM were excluded. So, the study can be comparable to that of former studies.

### 4.1 Limitations

First limitation of our study is that LNMs detected was dependent on the completeness of lymph node dissection and preoperative lymph node evaluation, which may lead to a partly degree of subjectivity. Nevertheless, thyroid and lymph nodes operations of DTC patients were performed by senior and experienced doctors to ensure that the metastatic lymph nodes can be removed as many as possible. Second concern is the retrospective evaluation of data.

### 4.2 Conclusion

With classification tree, the study found that LNMs and LNR could predict initial response to RAI, and their optimal cutoff values were 5 and 0.30, separately.

## ETHICAL APPROVAL STATEMENT

The Institutional Review Board (IRB) of the Second Hospital of Shandong University approved this study. All procedures complied with the Declaration of Helsinki for research involving human subjects.

## CONFLICT OF INTEREST

The authors declare that they have no conflicts of interest.

## Data Availability

The data that support the findings of this study are available upon request from the corresponding author. The data are not publicly available due to privacy or ethical restrictions.
